# Pubertal Hormonal Changes and the Autonomic Nervous System: Potential Role in Pediatric Orthostatic Intolerance

**DOI:** 10.3389/fnins.2019.01197

**Published:** 2019-11-12

**Authors:** Kassandra E. Coupal, Natalie D. Heeney, Brooke C. D. Hockin, Rebecca Ronsley, Kathryn Armstrong, Shubhayan Sanatani, Victoria E. Claydon

**Affiliations:** ^1^Department of Biomedical Physiology and Kinesiology, Simon Fraser University, Burnaby, BC, Canada; ^2^Department of Pediatrics, BC Children’s Hospital, Vancouver, BC, Canada; ^3^Children’s Heart Centre, BC Children’s Hospital, Vancouver, BC, Canada

**Keywords:** puberty, syncope, orthostatic intolerance, vasovagal, POTS

## Abstract

Puberty is initiated by hormonal changes in the adolescent body that trigger physical and behavioral changes to reach adult maturation. As these changes occur, some adolescents experience concerning pubertal symptoms that are associated with dysfunction of the autonomic nervous system (ANS). Vasovagal syncope (VVS) and Postural Orthostatic Tachycardia Syndrome (POTS) are common disorders of the ANS associated with puberty that are related to orthostatic intolerance and share similar symptoms. Compared to young males, young females have decreased orthostatic tolerance and a higher incidence of VVS and POTS. As puberty is linked to changes in specific sex and non-sex hormones, and hormonal therapy sometimes improves orthostatic symptoms in female VVS patients, it is possible that pubertal hormones play a role in the increased susceptibility of young females to autonomic dysfunction. The purpose of this paper is to review the key hormonal changes associated with female puberty, their effects on the ANS, and their potential role in predisposing some adolescent females to cardiovascular autonomic dysfunctions such as VVS and POTS. Increases in pubertal hormones such as estrogen, thyroid hormones, growth hormone, insulin, and insulin-like growth factor-1 promote vasodilatation and decrease blood volume. This may be exacerbated by higher levels of progesterone, which suppresses catecholamine secretion and sympathetic outflow. Abnormal heart rate increases in POTS patients may be exacerbated by pubertal increases in leptin, insulin, and thyroid hormones acting to increase sympathetic nervous system activity and/or catecholamine levels. Given the coincidental timing of female pubertal hormone surges and adolescent onset of VVS and POTS in young women, coupled with the known roles of these hormones in modulating cardiovascular homeostasis, it is likely that female pubertal hormones play a role in predisposing females to VVS and POTS during puberty. Further research is necessary to confirm the effects of female pubertal hormones on autonomic function, and their role in pubertal autonomic disorders such as VVS and POTS, in order to inform the treatment and management of these debilitating disorders.

## Introduction

### Puberty Is Associated With Orthostatic Intolerance

Puberty is a period of adolescence in which a child undergoes rapid changes that affect physical and mental functioning in order to reach adult maturation. During this time many adolescents experience substantial fatigue, mood swings, and stress (Larson et al., 1980). These symptoms of puberty are well-known and not generally worrisome (Larson et al., 1980; Wheeler, 1991; Viner and Christie, 2005; Table 1). However, other physical symptoms can occur at the onset of puberty that reflect autonomic nervous system (ANS) dysfunction, compromising the homeostatic regulation of basic bodily functions (Palma et al., 2017; Table 2). For example, puberty is associated with an increased incidence of syncope (fainting: transient loss of consciousness and postural tone) or presyncope (near-fainting), particularly in females (Walsh, 2001).

**TABLE 1:** Typical features of puberty.

**Physical changes**	**Mental/Emotional changes**
Breast, penis, testicle development	Mood swings – aggression, emotional surges, bouts of crying
Body hair – appearance in armpits and pubic area	Changes in sleep patterns and fatigue
Growth spurts and weight gain	Changes in social behavior
Menstruation and menstrual symptoms – nausea, cramps, bloating, diarrhea, aching in upper thighs, headache, backache, stomach ache	Menstrual symptoms – changes in appetite
Widening of the hips/shoulders	Risk-taking/novelty-seeking behaviors
Increased subcutaneous fat distribution/muscle development	Cognitive development

*Typical physical and mental/emotional changes are shown. Adapted from Larson et al. (1980),Wheeler (1991), and Viner and Christie (2005).*

**TABLE 2:** Concerning features of puberty.

**Physical changes**
Dizziness and syncope upon standing
Exercise intolerance
Sweating abnormalities – hyperhidrosis or hypohidrosis
Digestive difficulties – loss of appetite, bloating unrelated to menstruation
Urinary problems – difficulty urinating, incontinence, incomplete bladder emptying
Sexual dysfunction
Vision problems – blurred vision or inability for pupils to react to light quickly

*Concerning autonomic abnormalities associated with puberty are shown. Adapted from Palma et al. (2017).*

**FIGURE 1:**
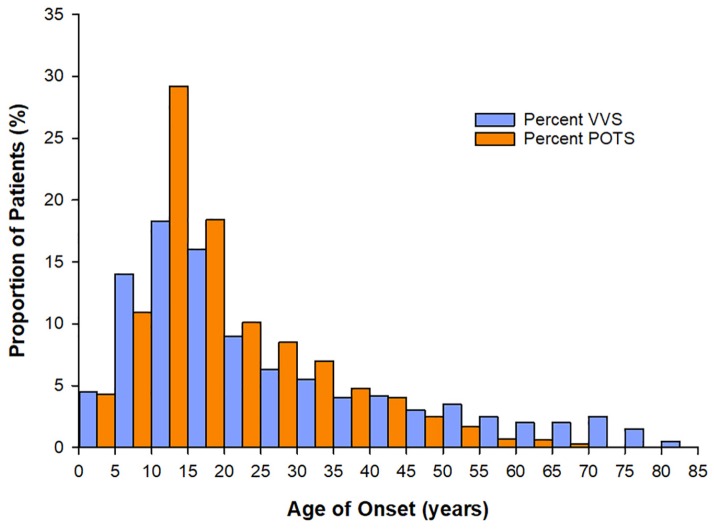
Age at onset of vasovagal syncope (VVS) and Postural Orthostatic Tachycardia Syndrome (POTS). For both patients with VVS (*n* = 443) and POTS (*n* = 4835) the peak age of onset of symptoms is between 10–15 years – coinciding with the age of onset of puberty. Data sourced from Kenny et al. (2010), Shaw et al. (2019).

Syncope and presyncope are common across the lifespan, but have a particularly high incidence in adolescents, with a peak age of onset during puberty at age ∼10–15 years (Figure 1); approximately 1 in three adolescents with syncope experience recurrent and severe episodes (de Jong-de Vos van Steenwijk et al., 1995; Driscoll et al., 1997; McLeod, 2003; Kenny et al., 2010; Kanjwal and Calkins, 2015). However, this prevalence is likely underestimated because many do not report their symptoms (Wieling et al., 2004; Ganzeboom et al., 2006; Nordkamp et al., 2009). Episodes are often related to impaired autonomic function, and are associated with anxiety, fatigue, headaches, dizziness, abdominal discomfort, nausea, and weakness, with significant impairments in quality of life (Braune et al., 1999; Rose et al., 2000; Radtke et al., 2011; Anderson et al., 2012; Armstrong et al., 2017). In pediatric populations, morbidity is equivalent to patients with asthma, end-stage renal disease and structural heart disease (Linzer et al., 1991; Anderson et al., 2012). Recurrent episodes are associated with injury due to falls secondary to loss of postural control, and may indicate more widespread autonomic abnormalities (Hainsworth et al., 2012). Affected children find these episodes distressing and exhibit sleep disturbances and difficulty concentrating, attending and focusing at school (Carapetian et al., 2008), as well as problems with exercising and participating in activities of daily living (Braune et al., 1999; Rose et al., 2000; Radtke et al., 2011; Anderson et al., 2012; Raj, 2013; Armstrong et al., 2017). The burden on healthcare resources is also substantial, with frequent medical and emergency visits (Kanjwal and Calkins, 2015) and extensive investigation – up to 35% see 10–20 physicians before diagnosis (Armstrong et al., 2017) and 10% of individuals still do not have a diagnosis 1 year after presenting in clinic (Van Dijk et al., 2007). Given their high incidence, marked healthcare burden, and severe impact on quality of life, a better understanding of the predisposing factors to adolescent syncope and presyncope and the potential role for pubertal hormones is warranted.

Syncope has many causes, including structural heart disease, cardiac arrhythmia, and impaired orthostatic cardiovascular control (Hainsworth et al., 2012). Here we focus on orthostatic (postural) syncope and presyncope, the most common forms in children and adolescents (Hainsworth et al., 2012). The most common sub-type of orthostatic syncope associated with puberty is vasovagal syncope (VVS) (Da and da Silva, 2014), responsible for up to 80% of pediatric syncope cases (Massin et al., 2004). Another condition that often coincides with the onset of puberty and presents with similar symptoms to VVS is Postural Orthostatic Tachycardia Syndrome (POTS) (Stewart, 2009). Both these conditions are associated with orthostatic intolerance, where the ANS does not function properly during changes in position or orthostatic stress. In broad terms, VVS reflects an excessive decrease in blood pressure and/or heart rate during orthostasis (Medow et al., 2008), while POTS displays an excessive increase in heart rate with orthostatic stress, with variable changes in blood pressure (Low, 2014).

**FIGURE 2:**
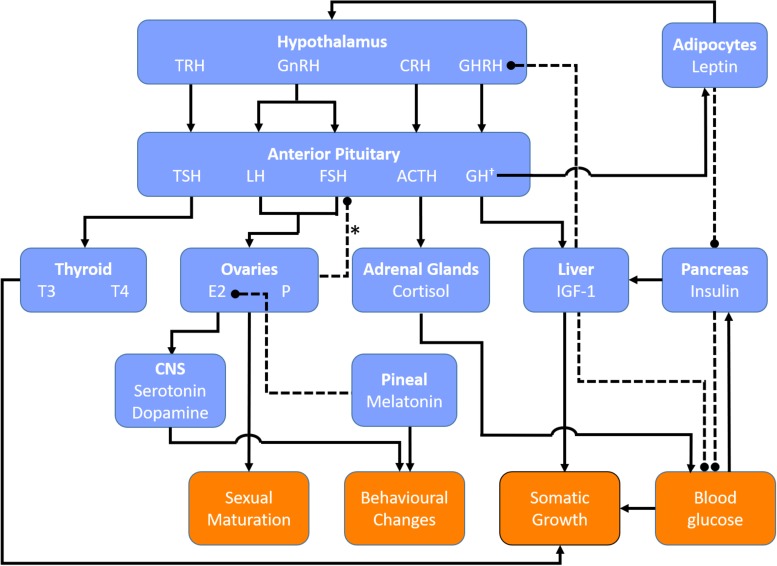
Key regulatory hormones involved in female puberty. Blue boxes denote hormones and their source of release (bold). Orange boxes denote end organ responses. Solid lines indicate positive feedback. Dashed lines indicate negative feedback. ^∗^Negative feedback from the ovaries on FSH secretion is primarily mediated via inhibins secreted by ovarian follicles. †GH secretion is stimulated by estrogen and thyroid hormones. ACTH, adrenocorticotrophic hormone; CRH, corticotropin releasing hormone; CNS, central nervous system; E2, estradiol; GH, growth hormone; GHRH, growth hormone releasing hormone; GnRH, gonadotropin releasing hormone; IGF-1, insulin-like growth factor-1; P, progesterone, TRH, thyrotropin releasing hormone; TSH, thyroid stimulating hormone; T3, triiodothyronine; T4, thyroxine.

The hormonal factors that initiate the onset and maintenance of puberty must be considered as possible culprits in the associated increased susceptibility to disorders of orthostatic intolerance, considering the timing of increased incidence of POTS and VVS with puberty (Kenny et al., 2010; Shaw et al., 2019; Figure 1). The initiation of puberty is prompted by a rise in activity of the hypothalamic-pituitary-gonadal (HPG) axis following a prolonged period of suppression during childhood (Forbes and Dahl, 2010). The HPG axis increases pulsatile release of gonadotropin-releasing hormones (GnRHs), stimulating gonadal hormones, and inducing various changes throughout the body to stimulate sexual maturation (Forbes and Dahl, 2010). Puberty is further associated with changes in other non-gonadal hormones such as GH, thyroid hormone, leptin, cortisol, and melatonin, which facilitate physical growth and behavioral changes in adolescents (Figure 2).

Females are known to have lower orthostatic tolerance compared to males (Meendering et al., 2005), with a 5:1 predominance of POTS (Low et al., 2009) and more than twice the incidence of syncope (Wieling et al., 2004). Since the initiation of puberty is prompted by a rise in activity of the HPG axis inducing changes in sex hormones (Clemans et al., 2010), it may be that some of the increased susceptibility to autonomic dysfunction in pubertal females could be attributed to hormonal changes occurring during this stage. Indeed, females with known VVS are reported to experience significant improvements in their symptoms with the introduction of hormonal therapy (Boehm et al., 1997). Orthostatic tolerance also improves in women across the lifespan, and is highest in postmenopausal females (Protheroe et al., 2013). These observations support the potential role of pubertal hormones in increasing susceptibility to cardiovascular autonomic dysfunction. Accordingly, the purpose of this review is to identify the key hormonal changes associated with female puberty, their effects on the ANS, and their potential role in predisposing adolescent females to cardiovascular autonomic dysfunctions such as VVS and POTS.

### Orthostasis Represents a Considerable Cardiovascular Challenge and Requires Compensation by the Autonomic Nervous System

Orthostasis is a common trigger for VVS and POTS because when a person is upright gravitational forces decrease arterial pressures in regions above the level of the heart, while simultaneously increasing lower body venous pooling and capillary filtration, reducing venous return (Hainsworth et al., 2012). If compensation for these hemodynamic consequences of orthostasis is inadequate, cardiac output is reduced and cerebral perfusion compromised, causing symptoms of presyncope that can progress to syncope (Hainsworth et al., 2012).

Orthostatic reductions in arterial pressure are sensed primarily by baroreceptors located in the aortic arch, coronary arteries, and carotid sinus (Hall and Guyton, 2011). The carotid sinus baroreceptors are particularly important in responding to orthostatic hemodynamic changes because of their location above the heart (carotid arterial pressure is about 15 mmHg lower than the pressure at the aortic root when upright, providing a potent stimulus to the carotid baroreceptors) (Hainsworth et al., 2012). Accordingly, during orthostasis these baroreceptors are unloaded, resulting in a reflex decrease in cardiac parasympathetic (vagal) tone, and increase in sympathetic outflow from the vasomotor center in the medulla to the heart and blood vessels (Hall and Guyton, 2011). The combined effect of these compensatory influences on the heart are increases in heart rate and contractility, accompanied by sympathetically mediated vasoconstriction of the resistance and capacitance vessels in the splanchnic, musculocutaneous, and renal vascular beds (Smit et al., 1999; Hall and Guyton, 2011). These coordinated adaptations are, therefore, associated with increases in total peripheral resistance, stroke volume, and blood pressure, with the maintenance of cardiac output and consequently cerebral perfusion (Medow et al., 2008). Given that these compensatory mechanisms are chiefly mediated by the ANS, impaired autonomic responses can predispose to loss of orthostatic control (Medow et al., 2008), leading to presyncope or syncope.

### Orthostatic Cardiovascular Responses Are Impaired in Patients With Vasovagal Syncope and Postural Orthostatic Tachycardia Syndrome

Ultimately, the cause of orthostatic presyncope or syncope is a failure of normal cardiovascular autonomic responses. However, different patterns of responses occur representing distinct autonomic syndromes, and this complicates their diagnosis and treatment. In POTS the primary problem is excessive orthostatic tachycardia, whereas in VVS it is impaired vasoconstriction and sudden hypotension, with or without reflex bradycardia or asystole (van Lieshout et al., 1991; McLeod, 2003; Hainsworth, 2004; Brignole, 2005; Mathias et al., 2012). These abnormalities can be subdivided further and may even coexist (Kurbaan et al., 1999; Brignole et al., 2000; Schroeder et al., 2011); however, the underlying mechanisms of these disorders remain unclear, particularly in children and adolescents.

Postural Orthostatic Tachycardia Syndrome is defined as “the development of orthostatic symptoms associated with a HR increment ≥30 bpm (beats per minute) [≥40 bpm in children (Singer et al., 2012; Zhao et al., 2015)], usually to ≥120 bpm [≥125 bpm in children aged 13–18 years or ≥130 bpm in children aged 6–12 years (Singer et al., 2012; Zhao et al., 2015)] without orthostatic hypotension” (Low et al., 2009). It is not clear what drives the change in cardiac responsiveness and the precise age at which this change occurs is unclear; pediatric POTS has been defined for chronological ages 12 years and younger (Kurbaan et al., 1999), but this definition does not reflect physiological age or pubertal status.

Postural Orthostatic Tachycardia Syndrome includes at least four subtypes: hyperadrenergic POTS with excessive sympathetic discharge; hypovolemic POTS (with compensatory tachycardia); neuropathic POTS with impaired vasoconstriction and compensatory tachycardia; and, rarely, noradrenaline transporter deficiency with synaptic noradrenaline accumulation (Low et al., 1995, 2009; Raj, 2013). Hemodynamic subtypes of VVS have also been recognized, and are characterized according to the relative contribution of hypotension and/or bradycardia to the event (Brignole et al., 2000). These distinctions provide mechanistic insight and may inform treatment, but present similarly, so are difficult to distinguish clinically. Whether adolescence is a time of general autonomic imbalance, and whether susceptibility to VVS and POTS are related to adolescence or pubertal hormone changes is unknown.

We and others demonstrated that in both patients with POTS and VVS, excessive venous pooling or capillary filtration (Brown and Hainsworth, 1999; Stewart et al., 2004), thermoregulatory vasodilation (Wilson et al., 2006; Hainsworth et al., 2012), low plasma or blood volumes (El-Sayed et al., 1995; Hoeldtke et al., 1995; El-Sayed and Hainsworth, 1996; Jacob et al., 1997; Mtinangi and Hainsworth, 1998, 1999; Lagi et al., 2003; Claydon et al., 2004), abnormal baroreflex responses (Thomson et al., 1997; Furlan et al., 1998; Gulli et al., 2001, 2005a,b; Cooper and Hainsworth, 2002), concurrent hypocapnia (Novak et al., 1998; Blaber et al., 2001; Carey et al., 2001; Lagi et al., 2001; Gisolf et al., 2004), excessive vascular responses to hypocapnia (Norcliffe-Kaufmann et al., 2007), and impaired cerebral autoregulation (Daffertshofer et al., 1991; Grubb et al., 1991; Claydon and Hainsworth, 2003) all increase susceptibility to orthostatic syncope in adults. In adults with VVS there is some evidence that the hypotension and reduced vascular resistance during hemodynamic collapse at presyncope may not be due to blunted sympathetic nerve activity *per se* but rather to other competing vasodilatory influences at that time (Vaddadi et al., 2010). There may also be an autoimmune component to susceptibility to both POTS and VVS in adults (Etienne and Weimer, 2006; Li et al., 2015; Ruzieh et al., 2017). These mechanistic insights have been central to the development of tailored management for affected adults; however, contributing mechanisms in children are less clear.

There may be a role for early excessive orthostatic cardiac sympathetic activation, and yet blunted vasoconstriction in children with POTS and VVS (Wieling et al., 1997; Moak et al., 2002; Laranjo et al., 2015; Wagoner et al., 2016), suggesting a disconnect between sympathetic outflow and the effector organ response. Indeed, some forms of POTS reflect selective neuropathy, with sympathetic denervation and impaired vascular resistance responses affecting the lower limbs (Raj, 2013). In children with VVS, initial increases in sympathetically–mediated vascular resistance responses are not sustained, and in fact abruptly reverse, precipitating hypotension (Moak et al., 2002). Orthostatic vasopressin and aldosterone are increased in children with VVS (Wagoner et al., 2016), presumably to compensate for impaired sympathetically–mediated vasoconstriction. Children with VVS and some forms of POTS have excessive venous pooling, particularly in the splanchnic vasculature (Stewart et al., 2004, 2006). Vitamin B12 deficiency (Wieling et al., 1997), low ferritin, and iron deficiency are reported in children with syncope (Antiel et al., 2011; Guven et al., 2013; Jarjour and Jarjour, 2013), presumably contributing to symptoms through anemia and low blood volumes.

Regardless of the underlying mechanism, orthostatic symptoms in children are associated with decreased cerebral blood flow velocity (Sung et al., 2000). Whether this is due to impaired cardiovascular control and compromised cerebral perfusion, or primary abnormalities in cerebral autoregulation is unclear. Interestingly, in adults with VVS cerebral pressure autoregulation is impaired (Claydon and Hainsworth, 2003) and combined with orthostatic hyperventilation (via activation of the respiratory muscle pump), with subsequent hypocapnia and cerebral vasoconstriction (Novak et al., 1998; Blaber et al., 2001; Carey et al., 2001; Lagi et al., 2001; Gisolf et al., 2004) that is compounded by increased cerebral reactivity to hypocapnia (Norcliffe-Kaufmann et al., 2007). The role and relative contribution of hypocapnia and cerebral autoregulation in pediatric syncope remain unclear.

Another contributor to most forms of orthostatic syncope in adults is hypovolemia, which may be associated with impaired renal sodium reabsorption in patients with POTS (Raj, 2013). Plasma or blood volume expansion improves orthostatic tolerance in adults (Rosen and Cryer, 1982; El-Sayed et al., 1995; Hoeldtke et al., 1995; El-Sayed and Hainsworth, 1996; Jacob et al., 1997; Mtinangi and Hainsworth, 1998, 1999; Lagi et al., 2003; Claydon et al., 2004; Cooper and Hainsworth, 2008), so it is possible that low plasma volumes also contribute to orthostatic syncope in children with POTS or VVS.

Lastly, physical deconditioning of the heart due to conditions such as viral infections or chronic fatigue has also been recognized as a possible mechanism underlying POTS (Fu et al., 2010). There is an association between POTS and cardiac deconditioning, and this is thought to be the result of a decreased heart size and associated decrease in cardiac output (Fu et al., 2010). The reasons why adolescent females are particularly susceptible to POTS remain unknown but perhaps their lower cardiac mass compared to adolescent males is a contributing factor.

## Hormonal Changes During Female Puberty and their Influences on Cardiovascular Autonomic Control

There are many hormonal changes during puberty that may have implications for autonomic cardiovascular control. Certainly, puberty seems to be a time of considerable change in autonomic function and cardiovascular and cerebrovascular regulation, with increases in endothelial function (Deda et al., 2015), and reductions in high frequency heart rate variability (a marker of cardiac vagal tone) in healthy adolescents following puberty (Tanaka et al., 2000). Puberty is also associated with increases in blood pressure (Tanaka et al., 2000; Deda et al., 2015), although this occurs to a much lesser extent in females than in males (Moran et al., 2008), with associated decreases in arterial stiffness during puberty in females, but not in males (Ahimastos et al., 2003). Cerebral blood flow is higher in children than in adults, and decreases markedly during puberty, with the peak reduction occurring in mid-adolescence (aged 15–17 years) – coinciding with the timing of peak incidence of syncope (Satterthwaite et al., 2014). In females, there is a partial recovery of cerebral blood flow in late puberty such that in adulthood, cerebral blood flow is greater in females compared to males (Satterthwaite et al., 2014) – coinciding with a time at which the particularly high incidence of onset of syncope in female adolescents begins to decrease. Some of these alterations in cerebral blood flow may reflect alterations in carbon dioxide levels. Higher end tidal carbon dioxide levels (P_ET_CO_2_) act as a potent cerebral vasodilator, increasing cerebral blood flow (Norcliffe et al., 2002; Claydon and Hainsworth, 2003). Young women breathe with an increased minute ventilation compared to young males (White et al., 1983), thus resting P_ET_CO_2_ is significantly higher in young males relative to young females (Dhokalia et al., 1998). This may render young women more susceptible to cerebral hypoperfusion and syncope, particularly in the face of orthostatic activation of the respiratory muscle pump and further associated increases in ventilation.

### Qualification of Pubertal Stages in Females

Tanner staging is a universally accepted means of qualifying pubertal development, with five proposed stages described by written criteria and illustrations (Marshall and Tanner, 1970; Swerdloff and Odell, 1975; Table 3). Tanner stage I refers to the preadolescent stage, while Tanner stage V represents the mature state (Marshall and Tanner, 1970). Normal timing of puberty varies and a child’s chronological age is not necessarily an accurate measure of pubertal development. Therefore, where possible, hormone levels are compared based on biological maturation stages rather than age. Given that sex hormones fluctuate in females depending on the phase of the menstrual cycle, overall changes in pubertal hormones will be considered independently of menstrual timing. A summary of the key hormones involved in the regulation of female puberty is provided in Figure 2.

**TABLE 3:** The Tanner stages of puberty in females.

**Tanner stage**	**Description**
I	Preadolescent
II	Breast budding; early labial hair growth
III	Increased breast size with palpable glandular tissue; no separating of breast contours; moderately dark coarser labial hair over mons pubis
IV	Further enlargement of breasts with projection of areola above breast plane; lateral spread of pubic hair
V	Adult breast size and pubic hair distribution

*Typical stages of puberty in girls are defined according to breast development and distribution of pubic hair. Adapted from Swerdloff and Odell (1975).*

### Initiation of Puberty

**FIGURE 3:**
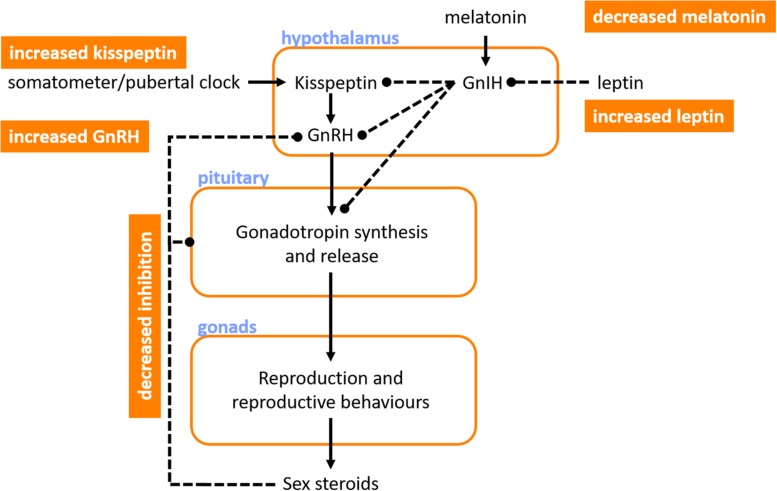
Key hormones involved in the initiation of puberty. Ultimately, puberty is initiated by profound increases in gonadotropin releasing hormone (GnRH) that initiate cyclic synthesis and release of gonadotropins, which then regulate reproduction, reproductive behaviors and secondary sexual characteristics, and sex steroid levels. Several factors may promote the increase in GnRH that precedes puberty, including: reduced sensitivity to inhibition of GnRH secretion by the sex steroids; increased leptin, which reduces inhibition of gonadotropins and their releasing hormones by the gonadotropin inhibiting hormone (GnIH); decreases in melatonin, with an associated reduction in GnIH production; and increases in kisspeptin, perhaps triggered by a “somatometer” or “pubertal clock,” which acts to further increase GnRH secretion. Orange boxes denote pubertal triggers. Blue text indicates sites of action or hormone release. Solid lines indicate positive feedback. Dashed lines indicate inhibitory influences.

Given the common coincidental timing of onset of symptoms of autonomic impairment and puberty, it is pertinent to consider the factors at play in initiating puberty, as well as the pubertal hormones that are involved as puberty progresses. The ultimate trigger for the onset of puberty is the initiation of profound increases in pulsatile GnRH secretion from the hypothalamus. The pulsatile nature of GnRH secretion with the onset of puberty is important, tonic GnRH administration does not induce luteinizing hormone (LH) or follicle stimulating hormone (FSH) secretion and so prevents ovulation (Marshall and Tanner, 1970). Numerous complementary mechanisms have been proposed to initiate the rise in pulsatile GnRH secretion (Figure 3). One key player is the loss of sensitivity to inhibition of GnRH secretion by ovarian sex steroids (such as estrogen and inhibins) (Gill et al., 2002; Shaw et al., 2010). Even very low levels of estrogen and inhibins block GnRH secretion in young children (Winter and Faiman, 1973). During puberty the levels of sex steroids required to block GnRH become progressively higher and this is permissive to increases in pulsatile GnRH, but not sufficient to trigger puberty (Messinis, 2006).

There may be a role for nutritional status and leptin in initiating puberty. Puberty starts earlier in overweight girls, and menstruation ceases with severe weight loss (Baker, 1985). Adiposity is linked to high leptin levels, and in animals leptin supplementation advances the onset of puberty compared to pair-fed animals (necessary because of the impact of leptin on appetite) – with leptin being permissive but not sufficient for the initiation of puberty (Cheung et al., 2001). This permissive role is likely via the indirect action of leptin (mediated via decreases in the antigonadotropic hormone, GnIH) on kisspeptin-expressing neurons that regulate GnRH secretion from the hypothalamus (Cunningham et al., 1999; Rhie, 2013). The arcuate nucleus contains abundant kisspeptin, a protein that is encoded by the Kiss1 gene, a GnRH pulse generating gene (Avendaño et al., 2017). Release of kisspeptin is inhibited until puberty when it rises and initiates increased pulsatile GnRH secretion. While it is likely that kisspeptin plays a major role in initiating puberty, the trigger for increased kisspeptin is unclear, with regulation suggested through either an internal “pubertal clock,” or a “somatometer” that monitors somatic (perhaps skeletal) development and triggers kisspeptin secretion once a key developmental threshold is reached (Avendaño et al., 2017).

Melatonin secretion from the pineal gland also seems to regulate pubertal onset. Melatonin release occurs during sleep and darkness, with higher secretion during the winter when there are reduced daylight hours (Brainard et al., 1982). The human pineal gland produces a substance (s) that keeps sexual maturation in check, which may be melatonin and/or GnIH (Silman et al., 1979). Indeed, destructive tumors of the pineal gland are associated with precocious puberty, and hypersecretory tumors with delayed puberty (Kitay, 1954; Silman et al., 1979). At the onset of puberty, melatonin levels decrease and this is associated with initiation of pubertal development (Silman et al., 1979). Indeed, in children living near the equator, who have lower levels of melatonin because of the long daylight hours, puberty occurs earlier than in those at higher latitudes (Dossus et al., 2013), perhaps reflecting the role of melatonin in initiation of puberty (Murcia et al., 2002). Interestingly, the onset of pulsatile secretion of GnRH during puberty initially occurs only during rapid-eye-movement sleep (Shaw et al., 2015). The stimulus for this is unknown, but may involve nocturnal melatonin secretion or possibly a genetically programed state of maturity of GnRH secreting neurons – evidence for the latter is not yet available, but has been hypothesized given the strong correlation between the age of menses onset between mother and daughter (Kolarov et al., 2005).

Many of these key hormonal cues thought to be involved in the initiation of puberty also continue to be involved as puberty progresses, largely through their role in influencing the HPG axis.

### The Hypothalamic-Pituitary-Gonadal Axis

**FIGURE 4:**
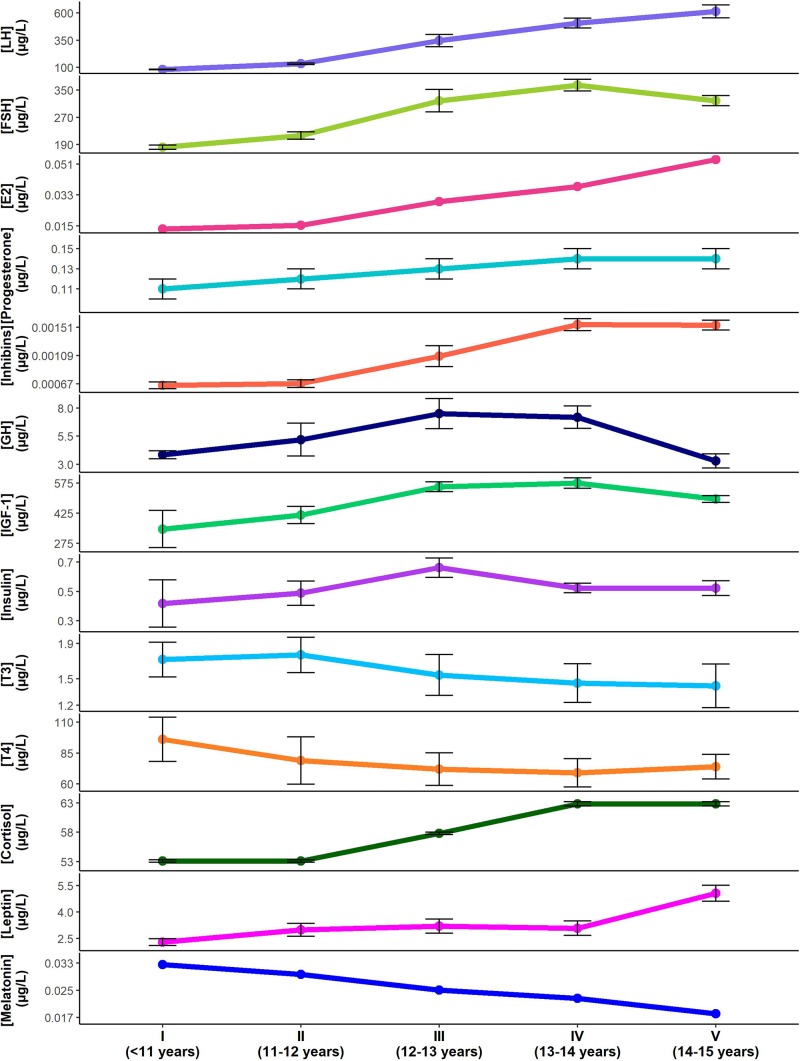
Changes in pubertal hormone levels according to Tanner stage and approximate age (mean ± standard error). Hormone concentrations for luteinizing hormone (LH) (Burger et al., 1988). follicle stimulating hormone (FSH) (Burger et al., 1988), Estradiol (E2) (Wennink et al., 1990), progesterone (Apter, 1980), inhibins (Wennink et al., 1990), growth hormone (GH) (Rose et al., 1991), insulin-like growth factor-1 (IGF-1) (Moran et al., 2002), insulin (Moran et al., 2002), triiodothyronine (T3) (Elmlinger et al., 2001), thyroxine (T4) (Elmlinger et al., 2001), cortisol (Stroud et al., 2011), leptin (Ahmed et al., 1999), melatonin (Crowley et al., 2012) are shown through the Tanner stages for females. Note that standard errors were not available for estradiol or melatonin data. Standard errors for LH, FSH and inhibins were approximated based on data provided. Cyclical changes in hormone levels with menstruation are not reflected.

Once puberty is initiated and the HPG axis is activated, GnRH is released from the hypothalamus (Figure 2), with peak levels at the onset of menstruation, after which its release becomes cyclical according to the phase of the menstrual cycle (Limonta et al., 2018). GnRH acts on the anterior pituitary to stimulate increases in LH and FSH production that induce sexual dimorphic changes in appearance as well as characteristic female behaviors (Limonta et al., 2018). The recent discovery of GnRH receptors in the ovary and endometrium raises the possibility of a role for GnRH outside of its hypothalamic functions (Limonta et al., 2018). LH and FSH levels increase with increases in GnRH pulse frequency and pulse amplitude during puberty. Levels of LH and FSH continue to increase until stage V for LH and stage IV for FSH, initially starting with undetectable levels of LH in prepubertal girls at stage I while low levels of FSH are detectable at this stage (Burger et al., 1988; Figure 4). During puberty, LH and FSH stimulate sex hormone secretion and regulate the menstrual cycle. LH acts on the ovaries to produce estrogen and facilitate egg maturation, while FSH is involved in follicle development and estrogen production (Limonta et al., 2018). LH also indirectly increases progesterone levels, secreted from the corpus luteum of mature follicles. Both LH and FSH appear to have indirect effects on the ANS, largely mediated through their effects on circulating estrogen and progesterone levels.

### Estrogen

Increased activity of the gonads stimulated by LH and FSH during puberty results in an associated increased production of estrogen. During puberty, estrogen levels initially mirror LH and FSH changes, with the lowest levels in prepubertal girls at stage I (Wennink et al., 1990; Figure 4). However, similar to LH, estrogen levels continue to increase until stage V, during which time FSH levels begin to decline (Wennink et al., 1990). Estrogen is a sex steroid hormone that is secreted by the ovary and binds to estrogen receptors (Wennink et al., 1990). It contributes to breast, vaginal, and uterine development, as well as female fat distribution, linear growth velocity, and skeletal maturation (Swerdloff and Odell, 1975). In this review we only consider 17-β estradiol (E2) because this is the most abundant, active, and best studied form of estrogen (Rybaczyk et al., 2005; Lasiuk and Hegadoren, 2007). Other natural estrogens, estrone (E1), and estriol (E3), have weak estrogenic properties and must be converted to E2 to have full estrogenic action; accordingly, they will not be considered further (Lasiuk and Hegadoren, 2007).

#### Estrogen Promotes Vasodilation and Hypocapnia, and Reduces Plasma Volume

Estrogen acts to acutely regulate vasomotor tone through endothelium-dependent and independent mechanisms (Mendelsohn and Karas, 1999; Maranon and Reckelhoff, 2013). E2 directly inhibits the influx of extracellular calcium into vascular smooth muscle via L-type calcium channels, preventing contraction and promoting vasodilatation (Mendelsohn and Karas, 1999). E2 also stimulates the opening of calcium-activated potassium channels through the nitric oxide (NO) and cyclic guanosine monophosphate-dependent pathways, relaxing smooth muscle and promoting vasodilatation (Mendelsohn and Karas, 1999). Lastly, E2 promotes rapid release of NO (Mendelsohn and Karas, 1999) and hydrogen sulfide (Dous et al., 2014), both of which are potent vasodilators. However, in addition to the acute effects on vasomotor tone, E2 also promotes chronic vasodilation through increased expression of endothelial nitric oxide synthase (eNOS), the enzyme that converts L-arginine to L-citrulline and NO, with a consequent vasodilatory response (Mendelsohn and Karas, 1999). In addition to promoting vasodilatation, E2 also acts to decrease plasma renin concentrations and angiotensin-converting-enzyme (ACE) with consequent reductions in plasma volume, accompanied by suppression of renal sympathetic activity, and ion and water reabsorption (Mendelsohn and Karas, 1999; Maranon and Reckelhoff, 2013). E2 also reduces circulating levels of the vasoconstrictor endothelin-1 (Mendelsohn and Karas, 1999). The net effect of these E2-mediated increases in vasodilatation and decreases in vasoconstriction and plasma volume, is a reduction in blood pressure, with the largest impact during stage V when E2 peaks. Interestingly, E2 enhances carotid vasomotor baroreflex sensitivity (but not cardiac baroreflex sensitivity), although this effect is likely mitigated by concurrent changes in progesterone levels, which blunt vasomotor baroreflex sensitivity (Brunt et al., 2013). Of note, estrogens have also been shown to increase cerebral blood flow in both animals and humans (Shamma et al., 1992; Belfort et al., 1995; Nevo et al., 2007), and loss of estrogens during menopause is associated with decreased cerebral reactivity (Matteis et al., 1998). The impact of vasodilation in the cerebral circulation secondary to increases in estrogen appears to be blunted in the face of estrogen and progesterone induced increases in respiration and hypocapnia, which would tend to reduce cerebral blood flow (Slatkovska et al., 2006; Preston et al., 2009).

#### Estrogen Has Indirect Effects on Central Nervous System Modulators That Regulate Behavior and Cardiovascular Control

Estradiol may also play a key role in regulating central nervous system factors that modulate adolescent behavior. For example, E2 may have the ability to modulate dopamine neurotransmission [dopaminergic neurons express estrogen receptors and mRNA (Purves-Tyson et al., 2012; Sinclair et al., 2014)], contributing to changes in dopamine signaling during adolescence (Sinclair et al., 2014). Dopamine is a catecholamine synthesized in dopaminergic neurons arising from the substantia nigra pars compacta and the ventral tegmental area and binds to dopamine receptor 1 (DR1) (Sinclair et al., 2014). Adult levels of DR1 mRNA and protein are attained during late adolescence/early adulthood, with coincidental timing to E2 levels (Sinclair et al., 2014). Dopamine influences control of movement, the ability to experience pleasure and pain, and emotional responses (Arain et al., 2013). In addition, dopamine inhibits noradrenaline release, and so acts to further amplify the vasodilatory effects of E2.

Increases in E2 have also been shown to increase serotonin concentrations by increasing the rate-limiting step in serotonin synthesis, as well as increasing the time that serotonin remains in the synapse and interstitial space (Rybaczyk et al., 2005). The latter is accomplished through the antagonistic action of E2 on the serotonin reuptake transporter (SERT) and down-regulation of SERT gene expression (Rybaczyk et al., 2005). Serotonin is an amine known to act as a neurotransmitter that is synthesized from tryptophan in serotoninergic neurons (Rybaczyk et al., 2005). Serotonin plays a role in arousal, anxiety, mood alterations and has also been shown to regulate various physiological functions including vasodilation (Arain et al., 2013). Thus, as E2 levels increase during female puberty, it is likely that these CNS modulators follow a similar increase, accounting for changes in behavior, and potentially exacerbating the vasodilatory effects of estrogen throughout puberty.

### Progesterone

Progesterone secretion generally occurs in conjunction with E2, as it is regulated by gonadotropins and secreted by the ovaries and the placenta (Rossmanith et al., 1990). It binds to progesterone receptors to thicken the lining of the uterus and stimulate the formation of milk glands in the breast in preparation for pregnancy (Regidor, 2014). Progesterone remains at fairly low levels during childhood until adolescence where it is shown to increase in a cyclic manner depending on the phase of the menstrual cycle (Apter, 1980; Figure 4). Progesterone levels continue to increase and peak premenopausally, declining thereafter until reaching very low levels after menopause (O’Connor et al., 2009).

#### Progesterone Causes Vasodilatation, Increases Plasma Volume and Promotes Hypocapnia

The effects of progesterone in the absence of estrogen indicate that it promotes vasodilatation, blunts sympathetic outflow and increases plasma volume (Brunt et al., 2013). Progesterone stimulates NO synthesis through transcriptional and non-transcriptional pathways, promoting vasodilation (Miner et al., 2011). However, progesterone also acts to counteract the effects of estrogen on NO production, resulting in mixed evidence concerning the net role of progesterone in controlling vasomotor tone (Brunt et al., 2011). Progesterone also blunts carotid-vasomotor baroreflex sensitivity (Brunt et al., 2013), but without affecting the control of mean arterial pressure, perhaps because of concurrent augmentation of baroreflex control of stroke volume and/or the opposing action of estrogen on baroreflex sensitivity (Brunt et al., 2011, 2013). Further research is needed to determine the precise role of progesterone in modulating vasomotor tone. However, the reported blunting of sympathetic outflow, which could account for decreases in heart rate and impaired vasoconstriction, may become relevant, particularly later in puberty.

Progesterone is a respiratory stimulant, with the consequence that P_ET_CO_2_ levels are reduced during times of high progesterone levels. For example, ventilation is increased during the luteal phase compared to the follicular phase of the menstrual cycle (White et al., 1983). This effect is enhanced during combined increases in both estrogen and progesterone (although estrogen alone is not closely correlated with cyclic fluctuations in ventilation during the menstrual cycle) (Regensteiner et al., 1989). Accordingly, higher estrogen and progesterone levels in younger women might contribute to their lower resting P_ET_CO_2_ in early life, and the age-related loss of estrogen would explain higher levels of P_ET_CO_2_ in later life (Regensteiner et al., 1989). These respiratory effects of estrogen and progesterone would be expected to promote reductions in P_ET_CO_2_ during puberty in females, particularly during the later stages, and might contribute to the reductions in cerebral blood flow that occur during female puberty.

### Inhibins

Inhibin levels are increased at puberty due to increased FSH stimulation of the granulosa cells of ovarian follicles, which are the main source of circulating dimeric inhibins (Bergadá et al., 2001). During puberty, a progressive rise in inhibins accompanies an increased production of sex steroids (Bergadá et al., 2001) with the highest levels reached during stages IV–V (Burger et al., 1988; Figure 4). With the development of ovarian follicles, inhibin levels increase where they act largely to suppress FSH release (Bergadá et al., 2001). This negative feedback control of FSH secretion only occurs once adult inhibin levels are reached (Bergadá et al., 2001).

#### Inhibins Regulate Follicle Stimulating Hormone Levels and Indirectly Affect Estrogen Levels

Once adult inhibin levels are reached at around stage IV, inhibins indirectly modulate estrogen levels through negative feedback control of FSH (Bergadá et al., 2001), with a theoretical impact on cardiovascular regulation via estrogen (Figure 2). However, E2 continues to increase from stage IV to V, indicating that the role of inhibin on overall estrogen levels is small. Inhibins play a key role in regulating estrogen levels during the menstrual cycle, but do not appear to affect the overall estrogen levels during the stages of puberty. Direct effects of inhibins on cardiovascular regulation have not been demonstrated, although they may play a role in gestational hypertension and preeclampsia (Itoh et al., 2006).

### Growth Hormone

Growth hormone (GH) increases substantially during the growth spurt of adolescence. It is secreted by the anterior pituitary gland in response to stimulation by GH releasing hormone from the hypothalamus (Soliman et al., 2014). GH levels more than double during puberty (Saenger, 2003), attaining peak levels at stage III–IV in females (Rose et al., 1991; Soliman et al., 2014; Figure 4). GH functions to promote lipolysis, increase protein synthesis, regulate energy metabolism in liver, muscle and adipose tissue, and is a potent insulin antagonist (Sakharova et al., 2008). GH levels are exquisitely regulated, with secretion enhanced by estrogen and thyroid hormones, and further regulation by somatostatin, ghrelin, and insulin-like growth factor 1 (IGF-1) levels. Circulating GH levels also regulate GH secretion through negative feedback (Romero et al., 2012).

#### Growth Hormone Is a Vasodilator and Induces Insulin-Resistance

Growth hormone acts as a vasodilator through activation of an endothelium-dependent component involving the NO pathway (Napoli et al., 2003) to improve arterial compliance, flow mediated dilation, and endothelial function (Napoli et al., 2003). Given its vasodilatory effects, GH would be expected to contribute to blood pressure lowering, particularly during stage III–IV where GH levels reach their peak. However, GH also acts to antagonize the hepatic and peripheral effects of insulin on glucose metabolism, preventing insulin uptake and inducing insulin resistance, thus increasing circulating insulin levels (Palmeiro et al., 2012). Furthermore, GH has a significant influence on adipocyte metabolism, increasing adipokines such as leptin (Palmeiro et al., 2012). Accordingly, GH contributes to increases in both insulin and leptin, increasing their impact on autonomic cardiovascular regulation.

### Insulin-Like Growth Factor-1

Insulin-like growth factor-1 (IGF-1) production follows similar patterns to GH secretion during puberty; it is stimulated by GH in the liver and further enhanced by estrogen and thyroid hormones (Rozario et al., 2000). In females, IGF-1 levels peak during stage IV and are associated with increases in adiposity at this time (Moran et al., 2002; Figure 4). IGF-1 binds to the IGF-1 receptor and is a primary mediator of the actions of GH, promoting growth in almost every cell in the body by regulating cellular proliferation, differentiation and metabolism (Rozario et al., 2000).

#### Insulin-Like Growth Factor-1 Is a Vasodilator and Enhances Insulin Sensitivity

Insulin-like growth factor-1 induces vasodilation by enhancing NO and potassium channel activity, both of which reduce calcium release into vascular smooth muscle, blunting vasoconstriction (Conti et al., 2004). IGF-1 interacts with a tyrosine kinase membrane receptor that activates the serine/threonine kinase Akt signaling pathway, which in turn activates eNOS, increasing NO levels and promoting vasodilatation (Conti et al., 2004). By facilitating widespread vasodilatation, it is likely that IGF-1 reduces blood pressure, particularly during stage III–IV when IGF-1 levels peak. IGF-1 increases insulin sensitivity and prevents postprandial dyslipidemia by suppressing plasma free fatty acid levels, reducing fasting plasma triglyceride concentrations, and increasing oxidative and non-oxidative glucose metabolism (Conti et al., 2004). Contrary to GH, IGF-1 helps to restore insulin and leptin levels to normal values (Conti et al., 2004). Thus, increases in IGF-1 are likely the result of, not the cause of, insulin resistance in puberty (Kelsey and Zeitler, 2016).

### Insulin

During female puberty insulin levels generally coincide with changes in IGF-1 levels (Moran et al., 2002). Fasting insulin levels are highest in stage III, occurring one stage earlier than peak IGF-1 levels (Moran et al., 2002; Figure 4). Insulin is synthesized by beta cells in the pancreas following stimulation by blood glucose (Sliwowska et al., 2014)and primarily acts to facilitate cellular glucose entry for energy utilization and growth (Sliwowska et al., 2014). When this process is compromised, insulin resistance can develop, and if left untreated it can progress to type 2 diabetes mellitus.

Puberty is associated with a marked decrease in insulin sensitivity, on par with that seen during pregnancy. In otherwise healthy youth, insulin sensitivity reaches a nadir in mid-puberty (stage III) that recovers by stage V (Kelsey and Zeitler, 2016). In patients with POTS, increases in serum resistin have been documented, the significance of which is unclear, but it has been previously associated with insulin resistance (Bai et al., 2017). It is interesting to note that the decline in cerebral blood flow during puberty is tightly linked to the concurrent decreases in glucose metabolism (Satterthwaite et al., 2014).

In children with type 1 diabetes, profound insulin resistance associated with puberty is well documented, although effects on the ANS in these children are not well studied. In one study evaluating 73 diabetic children, abnormalities in heart rate variability (a marker of autonomic dysfunction) were correlated with poor glycemic control in pubertal children. This relationship was not seen in younger children (Massin et al., 1999).

#### Insulin Exhibits Opposing Roles in Regulating Vasomotor Tone

Insulin exhibits both central and peripheral effects on the ANS – insulin both promotes vasodilatation and prevents vasoconstriction, while also having the ability to stimulate sympathetic activity. Insulin diminishes arterial stiffness (Vehkavaara et al., 2000) and acts as a vasodilator by binding to an insulin receptor tyrosine kinase, activating the Akt pathway and further stimulating eNOS to increase NO production (Muniyappa et al., 2007). Insulin has further been shown to attenuate vascular smooth muscle contraction and decrease vasoconstrictor tone by inhibiting calcium influx (Muniyappa et al., 2007). In contrast, insulin can stimulate sympathetic activity and increase catecholamine levels (Muniyappa et al., 2007). The opposing roles of insulin essentially lead to little change in arterial diameter and blood pressure under normal circumstances in healthy individuals (Muniyappa et al., 2007). However, during acute increases in sympathetic nerve activity, different parts of the vascular tree respond differently to insulin, where distal arterioles vasodilate and proximal arterioles constrict (Muniyappa et al., 2007). This could contribute to enhanced venous pooling during sympathetic activation upon standing. Increases in sympathetic activity and catecholamine levels would tend to facilitate tachycardia.

### Thyroid Hormones

The thyroid gland produces triiodothyronine (T3) and thyroxine (T4), both of which are stimulated by thyroid-stimulating hormone from the anterior pituitary gland secondary to release of thyrotropin-releasing hormone from the hypothalamus (Shahid and Sharma, 2019). The release of T3 and T4 from the thyroid gland is influenced by growth factors and modulated by sex steroids in females (Dunger et al., 1990). During puberty T3 increases, peaking during stage II, while T4 decreases at the onset of puberty and continues to decrease before leveling off after stage IV (Dunger et al., 1990; Elmlinger et al., 2001; Figure 4). The decrease in T4 is likely a result of its conversion into T3 in the periphery, as T3 is the more active, bioavailable form of thyroid hormone (Jørgensen et al., 1994). These hormones act to increase and regulate basal metabolic rate, as well as to increase heart rate, cardiac output, and ventilation, with decreases in peripheral resistance (Shahid and Sharma, 2019). Additional effects on the central nervous system and skeleton are crucial to normal development and growth (Shahid and Sharma, 2019).

#### Thyroid Hormones Act as Vasodilators While Increasing Heart Rate to Preserve Blood Pressure

Higher levels of thyroid hormones increase metabolism and heat production stimulating hypothalamic reflex responses (Thomas, 1957). These hypothalamic responses initiate vasodilation of arterioles, largely in the skin, to increase blood flow and dissipate heat, while simultaneously increasing heart rate and stroke volume to maintain a constant blood pressure (Thomas, 1957). T3 enhances endothelium-dependent relaxation through a cyclic adenosine monophosphate-mediated increase in endothelium-derived hyperpolarizing factor (EDHF), as well as through NO-mediated relaxation via up-regulation of eNOS (Büssemaker et al., 2003). EDHF prevents calcium influx through voltage-gated calcium channels, inhibiting contraction of vascular smooth muscle. T4 directly inhibits vascular contraction by inhibiting calcium/calmodulin-related regulatory mechanisms (Ishikawa et al., 1989). Cerebral blood flow remains unchanged during increases in thyroid hormones (Thomas, 1957).

### Cortisol

Baseline cortisol levels increase during puberty, peaking at stage IV/V in females (Stroud et al., 2011; Figure 4). Cortisol is released by the adrenal gland following stimulation by adrenocorticotropic hormone, which is produced by the anterior pituitary (Lee et al., 2015). Cortisol binds to cortisol receptors and plays a role in maintaining blood glucose levels, central nervous system function, and cardiovascular function during fasting (Lee et al., 2015). Cortisol increases blood glucose levels during stress at the expense of muscle protein and one of its most important functions is to protect the body against self-injurious inflammatory and immune responses (Lee et al., 2015).

#### Cortisol Increases Plasma Volume and Increases Blood Pressure

Acute increases in cortisol levels blunt autonomic reactivity by suppressing the early effects of catecholamines in the brain (Teixeira et al., 2015), with subsequent decreases in arterial vasoconstriction (Theodorakis et al., 1998). However, chronic elevations in cortisol induce hypertension, independent of changes in sympathetic nervous system activity, likely mediated via increases in plasma volume, extracellular fluid and exchangeable sodium (Theodorakis et al., 1998).

### Leptin

Leptin levels increase with the onset of puberty, but remain fairly steady following this initial rise until a late surge starting at stage IV in females (Ahmed et al., 1999; Figure 4). Leptin is secreted by adipocytes and is hypothesized to act on specific receptors at the level of the hypothalamus to regulate appetite, energy expenditure, the neuroendocrine axis, and weight (Ahmed et al., 1999). Differential changes in body composition between males and females during puberty are affected by the sexual dimorphism of leptin levels during this period (Ahmed et al., 1999). In females, leptin increases throughout puberty, with a prominent surge during stage IV–V, to reach adult concentrations, while in males it increases only transiently with the onset of puberty (Kelsey and Zeitler, 2016).

#### Leptin Activates the Sympathetic Nervous System and Increases Insulin Sensitivity

Leptin activates the sympathetic nervous system through hypothalamic mechanisms that are mediated by neuropeptide systems, including the melanocortin system and corticotropin-releasing hormone (Muniyappa et al., 2007). With increases in sympathetic activity, increases in heart rate and vasoconstriction are expected, particularly at stage V, when leptin levels peak. Leptin also increases insulin sensitivity which helps to prevent abnormally high levels of circulating insulin, and somewhat counteracts the role of GH in inducing insulin-resistance (Muniyappa et al., 2007). Higher leptin levels are also associated with impaired heart rate variability (a sign of autonomic dysfunction). In particular, increased low frequency heart rate variability, and an increased ratio of low to high frequency heart rate variability have been reported, suggesting that increased leptin levels may result in an autonomic imbalance with a sympathetic predominance in young females during puberty (Van De Wielle and Michels, 2017).

### Melatonin

In the early stages of female puberty (I and II) melatonin secretion significantly decreases, with further successive decreases in the later stages (Murcia et al., 2002; Crowley et al., 2012; Figure 4). Melatonin is a lipophilic endocrine hormone that is synthesized in the pineal gland (Murcia et al., 2002). Melatonin secretion is largely regulated by light and dark information received by the suprachiasmatic nucleus from retinal photosensitive ganglionic cells (Murcia et al., 2002). It is secreted in a circadian pattern, with the greatest secretions occurring at night (Murcia et al., 2002), and functions in many regulatory processes including biological rhythms, metabolism, intestinal reflexes, and protection against inflammation (Chen et al., 2011).

#### Melatonin Inhibits Estrogen Receptor-Mediated Transcription

Melatonin interferes with E2 signaling by impairing estrogen receptor pathways via specific inhibition of E2-induced estrogen receptor alpha (ERα) mediated transcription of both estrogen response element and activator protein 1 containing promoters (Del Río et al., 2004). By reducing the transcription of one of the main estrogen receptors, ERα, the effects of estrogen are reduced. As melatonin levels decrease as puberty progresses, while estrogen increases, there is an inverse relationship between melatonin and estrogen. Thus, the inhibitory effect of melatonin on estrogen signaling is probably relatively minor during puberty.

### Kisspeptin and Gonadotropin Inhibiting Hormone

Increases in kisspeptin levels and decreases in GnIH levels are thought to contribute to the initiation of puberty (Figure 3). However, data on kisspeptin and GnIH levels throughout the phases of puberty, as well as any potential impact on cardiovascular responses or susceptibility to syncope are currently lacking.

## Pubertal Hormones as Potential Contributors to Vasovagal Syncope and Postural Orthostatic Tachycardia Syndrome

There are numerous factors known to increase susceptibility to syncope. One key contributor is excessive vasodilatation and/or impaired vasoconstrictor responses, often associated with hypotension and blunted sympathetic outflow (Brown and Hainsworth, 2000; Bush et al., 2000). In addition, hypocapnia and cerebral hypoperfusion (Claydon and Hainsworth, 2003; Norcliffe-Kaufmann et al., 2007), hypovolemia (Cooper and Hainsworth, 2008), excessive tachycardia (Sandroni et al., 1999), or impaired baroreflex responses (Cooper and Hainsworth, 2002; Gulli et al., 2005a), will all impair orthostatic cardiovascular control. The potential impact of female pubertal hormones on these predisposing factors is summarized in Table 4.

**TABLE 4:** Impact of pubertal hormones on factors that predispose to syncope events.

**Factors predisposing orthostatic intolerance**	**Pubertal hormones that exacerbate**	**Pubertal hormones that ameliorate**
Vasodilatation	Estrogen	Progesterone (by inhibiting action of estrogen)
	Progesterone	Melatonin (by inhibiting action of estrogen)
	Growth hormone	
	IGF-1	
	Insulin	
	Thyroid hormones	
Impaired vasoconstriction	Insulin	Leptin
	Progesterone	
Hypotension	Estrogen	Cortisol
	Growth hormone	
	IGF-1	
Low sympathetic activity	Progesterone	Leptin
		Insulin
Excessive venous pooling	Insulin	
Hypovolemia	Estrogen	Cortisol
		Progesterone
Hypocapnia	Estrogen	
	Progesterone	
Decreased cerebral blood flow	Estrogen and progesterone (via hypocapnia)	Estrogen
Excessive tachycardia	Insulin	Progesterone
	Leptin	
	Thyroid hormones	
Decreased baroreflex sensitivity	Progesterone	Estrogen

*IGF-1, insulin-like growth factor-1.*

**FIGURE 5:**
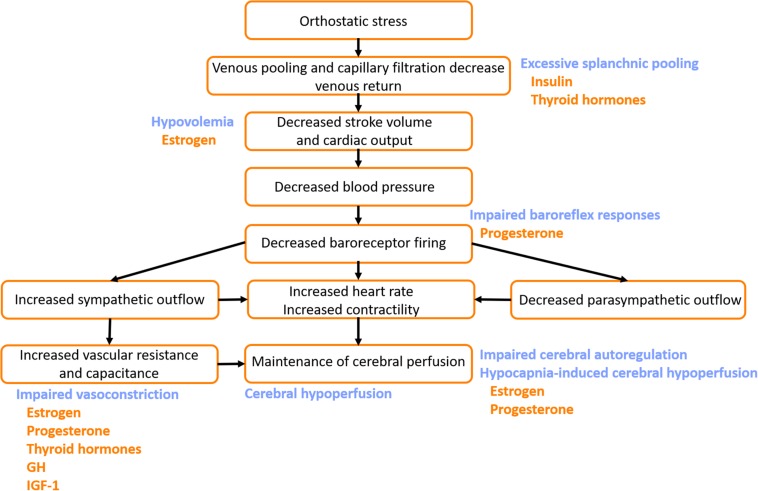
Potential mechanisms by which pubertal hormones exacerbate susceptibility to vasovagal syncope (VVS). The normal cardiovascular response to orthostatic stress is shown (black text). In patients with VVS, hypovolemia and excessive venous pooling, particularly in the splanchnic vascular bed, combine to produce particularly large orthostatic reductions in stroke volume and cardiac output. Impaired baroreflex responses, and blunted orthostatic increases in vascular resistance and vascular capacitance fail to appropriately compensate, and ultimately blood pressure and cardiac output fall dramatically, with cerebral hypoperfusion and subsequent presyncope or syncope. Impairments in cerebral autoregulatory control and excessive reductions in cerebral blood flow in response to the hypocapnia during orthostasis further contribute to the decline in cerebral perfusion (blue text). Hormones that potentially exacerbate the impaired cardiovascular responses to orthostatic stress in patients with VVS are indicated (orange text). GH, growth hormone; IGF-1, insulin-like growth factor-1.

**FIGURE 6:**
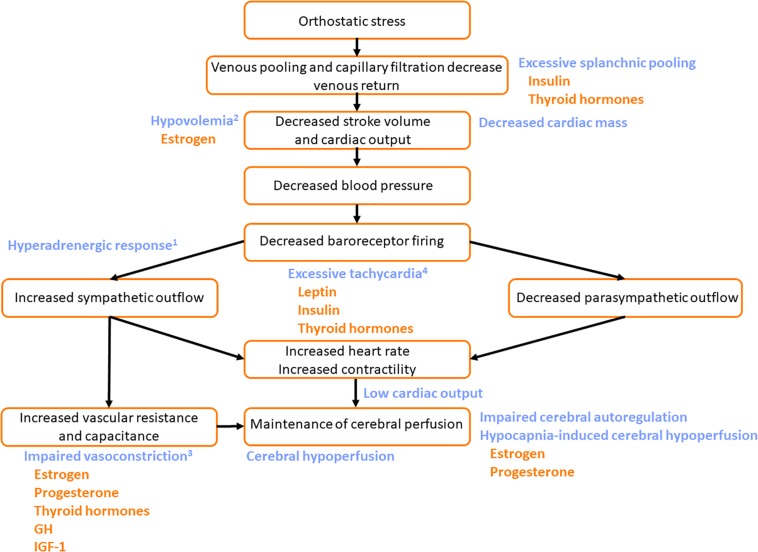
Potential mechanisms by which pubertal hormones exacerbate susceptibility to the Postural Orthostatic Tachycardia Syndrome (POTS). The normal cardiovascular response to orthostatic stress is shown (black text). In patients with POTS, excessive venous pooling, particularly in the splanchnic vascular bed, contributes to particularly large orthostatic reductions in stroke volume and cardiac output. In those with hyperadrenergic POTS^1^ central sympathetic outflow is excessive. In those with hypovolemic POTS^2^, plasma volume is low exacerbating the impact of the orthostatic venous pooling. Patients with neuropathic POTS have impaired vasoconstrictor responses with compensatory tachycardia^3^. Those with noradrenaline transporter deficiency have a primary orthostatic tachycardia^4^. The common feature of all subtypes is excessive tachycardia (either primary tachycardia or secondary tachycardia to compensate for other impairments) with low venous return and stroke volume during orthostasis, reduced time for diastolic filling, and consequently low cardiac output. Cerebral perfusion is compromised and symptoms of presyncope are common. Impaired orthostatic increases in vascular resistance and vascular capacitance exacerbate the orthostatic tachycardia. Some patients with POTS are reported to have decreased cardiac mass, with low stroke volume, that also facilitates orthostatic tachycardia. Impairments in cerebral autoregulatory control and excessive reductions in cerebral blood flow in response to the hypocapnia during orthostasis further contribute to the decline in cerebral perfusion (blue text). Hormones that potentially exacerbate the impaired cardiovascular responses to orthostatic stress in patients with POTS are indicated (orange text). Leptin, insulin and thyroid hormones may exacerbate orthostatic tachycardia. GH, growth hormone; IGF-1, insulin-like growth factor-1.

The implications for these hormones as possible contributors to VVS and POTS can be considered based on the impact each has on cardiovascular control (Table 4), and the timing of their changes over the duration of puberty (Apter, 1980; Burger et al., 1988; Wennink et al., 1990; Rose et al., 1991; Ahmed et al., 1999; Elmlinger et al., 2001; Moran et al., 2002; Stroud et al., 2011; Crowley et al., 2012; Figure 4). Hormones promoting vasodilatation may exacerbate the inappropriate reductions in blood pressure that occur during VVS or the impaired vasoconstriction in POTS, placing increased reliance on orthostatic tachycardia for maintenance of blood pressure (Figures 5, 6). Thyroid hormones, GH and IGF-1 largely act as vasodilators through their involvement with the NO pathway. Along with releasing NO and hydrogen sulfide, another vasodilator, E2 also promotes hyperpolarization in vascular smooth muscle, and decreases renal renin release with subsequent reductions in angiotensin-mediated vasoconstriction, culminating in a potent vasodilatory influence. Baseline blood pressures increase during puberty, but to a lesser degree in females than in males, suggesting that the vasodilatory actions of female pubertal hormones promote lower blood pressures in females, with an associated susceptibility to further orthostatic blood pressure decrements (Shankar et al., 2005). The differential influence of insulin in the presence of sympathetic stimulation may account for the abnormal blood perfusion and splanchnic pooling as a proposed mechanism of VVS and POTS (Figures 5, 6). In the presence of high insulin and sympathetic activity, as seen with standing, distal vessels vasodilate while proximal vessels constrict, likely increasing blood flow to distal extremities while decreasing flow to the central regions of the body. This effect would predominate during stage III when insulin levels peak. Similarly, different organs in the body respond differently to the vasodilatory effects of thyroid hormones, and this may also promote splanchnic pooling (Figures 5, 6). The presence of hypovolemia in POTS and VVS patients may be exacerbated by E2 as it acts to decrease renin concentrations with consequent decreases in blood volume mediated via the renin-angiotensin-aldosterone pathway. High levels of E2 and progesterone may promote hypocapnia and associated reductions in cerebral blood flow, increasing susceptibility to further cerebral compromise during orthostatic stress. While these hormones can account for some of the similar predisposing factors and symptoms of VVS and POTS, hormones that may contribute to the different profiles of VVS and POTS should further be considered.

The effects of these hormones on the ANS may be more prominent in some individuals, leading to a greater predisposition to POTS or VVS. As cortisol levels have been shown to increase during vasovagal reactions in patients with VVS (Stroud et al., 2011), peak levels of cortisol during stage IV/V may play a role in the manifestation of VVS. Progesterone may also be a contributor to VVS by blunting sympathetic outflow, and impairing baroreflex responses, exhibiting its greatest effects during stages IV–V, when progesterone is at its highest (Figure 5).

Central hyperadrenergic POTS and the abnormal heart rate increases in POTS patients could be exacerbated by hormones involved in increasing sympathetic activity and elevating norepinephrine levels (Figure 6). Both insulin and leptin have the ability to increase sympathetic activity and stimulate catecholamine release, and increases in leptin and insulin are associated with increases in heart rate. Insulin and leptin peak during puberty in stages III and V, respectively, and might be hypothesized to exacerbate orthostatic tachycardia at these times. Thyroid hormones also play a role in increasing heart rate to maintain blood pressure – with key rises in T3 during pubertal stage II.

While it is certainly not possible to imply causality between these pubertal hormone changes and disorders of orthostatic tolerance in adolescents, the coincidental timing of these profound hormonal changes – many of which have marked cardiovascular effects - and the timing of onset of VVS and POTS, together with the observation that many youth “grow out” of their symptoms (Kizilbash et al., 2014), is suggestive of a link between pubertal hormone changes and susceptibility to syncope in females. Certainly, orthostatic tolerance increases in women as they age, and is highest in postmenopausal women (Protheroe et al., 2013). It is conceivable that pubertal increases in these hormones, many of which have secondary actions to promote vasodilatation, impair vasoconstriction, decrease blood volume, promote hypocapnia and cerebral hypoperfusion, and contribute to excessive tachycardia, unmask a susceptibility to disorders of orthostatic intolerance in girls with a previously unknown predisposition to poor orthostatic tolerance. Further insight into the role of female sex hormones in susceptibility to VVS and POTS can also be gleaned from a case series in which fifteen women (including six adolescents) with refractory VVS/POTS experienced symptomatic benefit with complete resolution, or a marked reduction in the frequency, of orthostatic symptoms following ovarian hormone therapy (Boehm et al., 1997).

## Conclusion

Hormone changes during puberty have the potential to impact cardiovascular autonomic control and as such may play a role in predisposing adolescent females to autonomic dysfunction, including disorders of orthostatic intolerance such as POTS and VVS. The peak incidence of VVS and POTS in young women occurs at approximately 10–15 years of age, a time where many hormones involved in puberty, capable of predisposing to disorders of orthostatic intolerance, are at peak levels. These pubertal hormones can act to promote vasodilatation, impair vasoconstriction, decrease plasma volume, promote hypocapnia and cerebral hypoperfusion, and contribute to excessive tachycardia. Additional research is necessary to examine the potential role that puberty, and in particular the hormonal changes that accompany it, may have in predisposing young females to orthostatic intolerance and autonomic dysfunction during their pubertal years.

## Author Contributions

KC, NH, BH, and VC wrote the manuscript. RR, KA, and SS provided critical analysis, insight, editing, and review of intellectual content.

## Conflict of Interest

The authors declare that the research was conducted in the absence of any commercial or financial relationships that could be construed as a potential conflict of interest.
